# P-866. Optimizing Antimicrobial De-escalation in Sepsis Patients in ICUs with High Multidrug Resistance

**DOI:** 10.1093/ofid/ofaf695.1074

**Published:** 2026-01-11

**Authors:** Shakthivel Dhandapani, Sam Johnson Udaya Chander

**Affiliations:** Sri Ramakrishna Institution of Paramedical Sciences, College of Pharmacy, COIMBATORE, Tamil Nadu, India; Sri Ramakrishna Institution of Paramedical Sciences, College of Pharmacy, COIMBATORE, Tamil Nadu, India

## Abstract

**Background:**

Antimicrobial de-escalation is a vital aspect of empirical antimicrobial therapy, which is considered difficult in high antimicrobial resistance. This study aims to evaluate the effectiveness of antimicrobial de-escalation on the clinical outcomes of ICU sepsis patients with high antimicrobial resistance.

**Figure:**
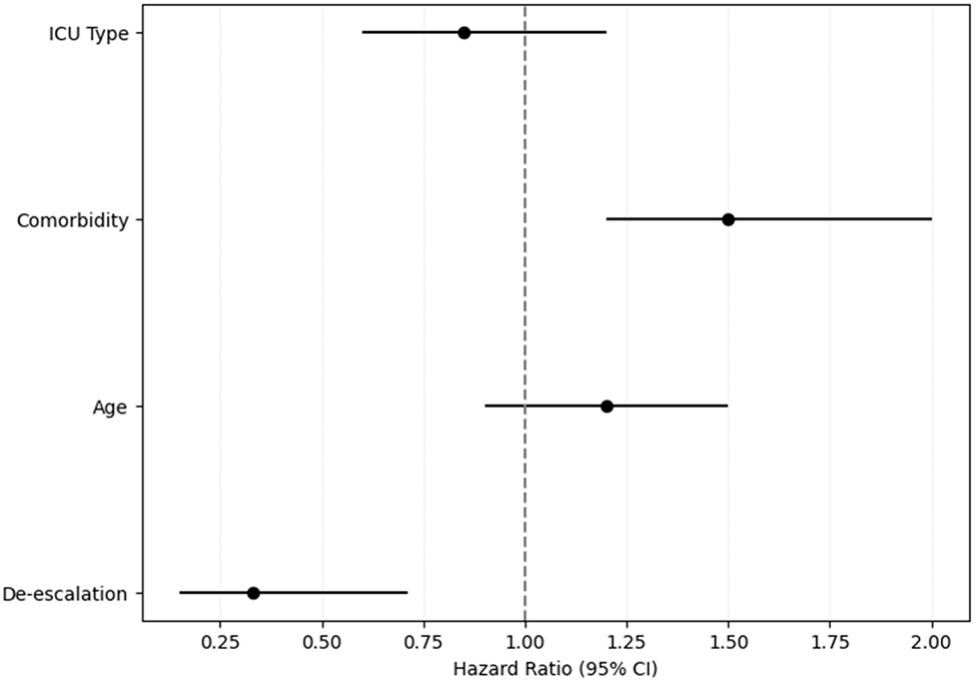
Forest Plot of Hazard Ratios from Cox Proportional Hazards Regression Analysis This forest plot shows the hazard ratios (HR) and 95% confidence intervals (CI) for key variables, including antimicrobial de-escalation, age, comorbidity, and ICU type. The vertical dashed line at HR = 1 represents no effect, with values to the left indicating reduced hazard. Antimicrobial de-escalation significantly reduces hazard (HR 0.33; 95% CI 0.15–0.71).

**Figure:**
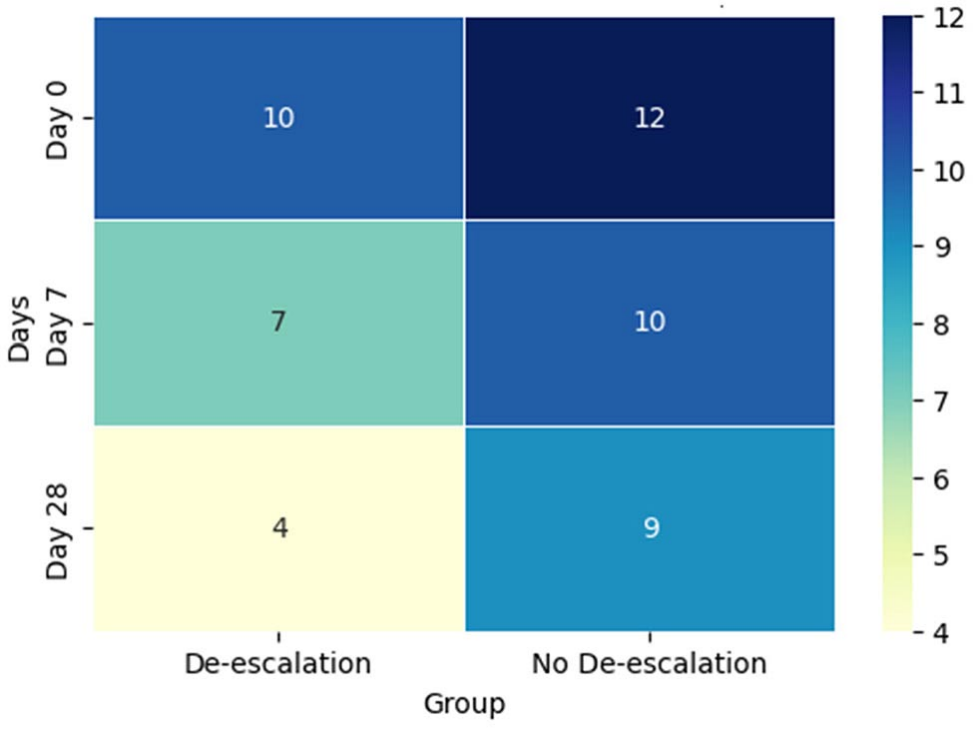
Heatmap of SOFA Scores Over Time in ICU Patients Receiving vs. Not Receiving Antimicrobial De-escalation Heatmap showing the progression of SOFA (Sequential Organ Failure Assessment) scores across three time points (Day 0, Day 7, Day 28) for ICU patients who received antimicrobial de-escalation therapy versus those who did not. The color intensity indicates the severity of organ failure, with lower scores representing less severe organ dysfunction. Patients receiving de-escalation therapy showed a reduction in SOFA scores over time, indicating a potential improvement in clinical outcomes.

**Methods:**

This prospective, observational, multicenter study included 260 sepsis patients admitted in ICUs of six tertiary care hospitals located in Coimbatore, India. Patients were categorized by the status of de-escalation therapy and matched according to SOFA scores to assess the severity of illness. Patients who received de-escalation therapy were compared to patients who did not receive de-escalation therapy, using a propensity score matching based on their SOFA score at initiation of de-escalation.

**Figure:**
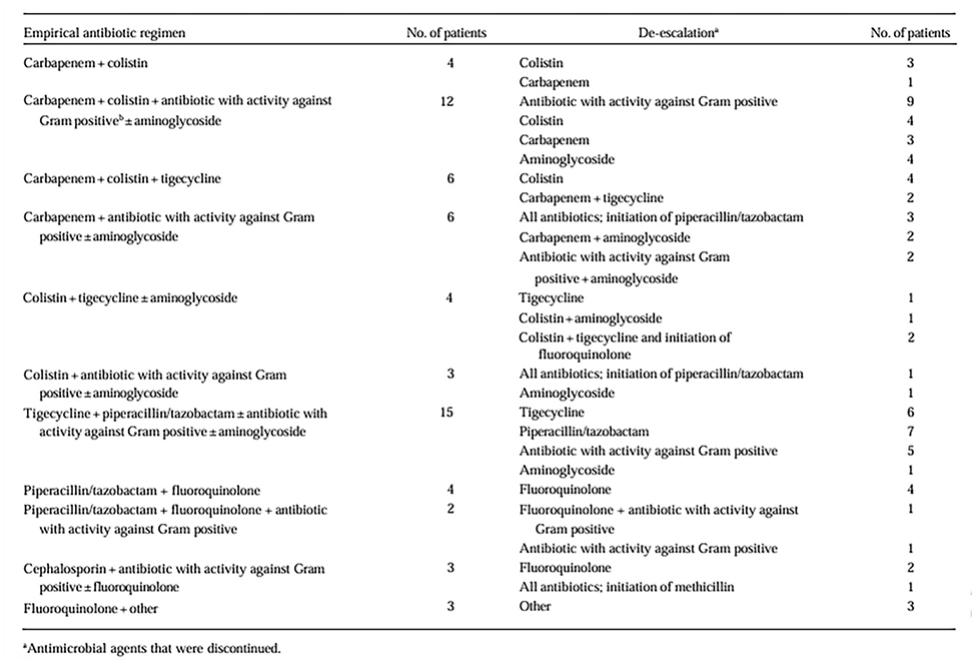
Empirical Antibiotic Regimens and De-escalation Practices in the De-escalation Group (n = 62) This table summarizes the initial broad-spectrum antibiotic regimens administered to ICU patients and the corresponding de-escalation strategies applied. Regimens frequently included combinations such as carbapenem with colistin or tigecycline. De-escalation commonly involved narrowing therapy to agents like fluoroquinolones, aminoglycosides, or monotherapy with targeted antibiotics, reflecting efforts to reduce antimicrobial pressure while maintaining clinical coverage.

**Results:**

A total of 260 patients (mean age 61.8 ± 14.9 years) were included in the study. Antibiotic-resistant pathogens accounted for 63% and were classified as 13% MDR, 48% extensively drug-resistant and 2% pandrug-resistant. In 96 cases (37%), the de-escalation strategy was not feasible based on antibiotic susceptibility test results. Of the remaining 164 patients eligible for de-escalation, this strategy was implemented to 62 patients (23.8%). The patients were matched with an equal number of patients who didn't undergo de-escalation. In the matched cohort of 124 patients, antimicrobial de-escalation was associated with a reduction in overall mortality (14% vs. 35.5%; OR 0.29, 95% CI 0.12–0.68; P = 0.007). De-escalation also resulted in significantly lower ICU and hospital mortality rates. Additionally, a decrease in SOFA scores was observed among patients who underwent de-escalation. Cox multivariate regression analysis further identified de-escalation as an independent predictor of improved 28-day survival (HR 0.33; 95% CI 0.15–0.71; P = 0.006).

**Conclusion:**

The de-escalation of antimicrobials, even though being limited by high levels of resistance, has also been linked with lower mortality and better outcomes for patients with sepsis when it is possible. These results suggest the use of antimicrobial de-escalation in medical settings as a reliable and effective approach to treatment of sepsis in situations with high resistance.

**Disclosures:**

All Authors: No reported disclosures

